# Patterns of individual coping, engagement with social supports and use of formal services among a five-country sample of resilient youth

**DOI:** 10.1017/gmh.2015.19

**Published:** 2015-11-03

**Authors:** M. Ungar, L. Theron, L. Liebenberg, Guo-Xiu Tian, A. Restrepo, J. Sanders, R. Munford, S. Russell

**Affiliations:** 1 Resilience Research Centre, Dalhousie University, Halifax, Canada; 2 Optentia Research Focus Area, Faculty of Humanities, North-West University, Vanderbijlpark, South Africa; 3 School of Social Work, Dalhousie University, Halifax, Canada; 4 Political Science, Capital Normal University, Beijing, China; 5 School of Public Health, University of Antioquia, Medellin, Colombia; 6 School of Social Work, Massey University, Palmerston North, New Zealand

**Keywords:** Coping, resilience, service use patterns, social support, young people

## Abstract

**Background.:**

Although resilience among victims of child abuse is commonly understood as a process of interaction between individuals and their environments, there have been very few studies of how children's individual coping strategies, social supports and formal services combine to promote well-being.

**Method.:**

For this study, we conducted a multi-phase analysis of a qualitative dataset of 608 interviews with young people from five countries using grounded theory strategies to build a substantive theory of young people's service and support use patterns. We started with an analysis of ten interviews (two from each country) and then compared these findings to patterns found in each country's full dataset.

**Results.:**

The substantive theory that emerged explains young people's transience between individual coping strategies (cognitive and behavioral), reliance on social supports (family members, peers and teachers), and engagement with formal service providers whose roles are to provide interventions and case management. Young people's patterns of navigation were shown to be contingent upon the individual's risk exposure, his or her individual capacity to cope, and the quality of the formal and informal supports and services that are available and accessible.

**Conclusion.:**

Differing amounts of formal resources in low-, middle- and high-income countries influence patterns of service use. Implications for better coordination between formal mental health services and social supports are discussed.

While the study of resilience has become increasingly popular, most research has concentrated on individual and relational characteristics of young people (e.g. self-efficacy, attachments, mentorship, family functioning and so forth) (Bonanno & Mancini, 2012). Although youth who are exposed to high levels of risk, including maltreatment, are often clients of multiple social and health services (Grimes *et al*. 2006; Ungar *et al*. 2012), there has been very few investigations of the importance of service delivery systems and their substitutes (e.g. informal supports like mentors in context where services are in short supply) on processes associated with resilience. Likewise, little of the research on resilience has explored why young people seek out and accept services or decline services that can improve their capacity to cope following abuse. The research reported in this paper addresses these gaps in knowledge.

Specifically, we wanted to answer the following questions: (1) What combination of services and supports do young people need for successful coping following experiences of maltreatment, exposure to violence or dislocation? (2) What factors influence these young people's engagement with formal and informal services and supports? and (3) In relatively highly stressed, resource-poor contexts (when compared to other contexts locally), how do young people who have experienced adversity cope if formal social and health services are not available or accessible? While individual coping strategies are known to be helpful to children in contexts of lower risk exposure (Ungar, 2015), child welfare and mental health services appear to exert a greater impact on outcomes in contexts of exposure to higher amounts of risk. It remains unclear from previous studies; however, whether children's rejection of formal programming is a healthy adaptation to what they perceive as failed services or a capacity-building self-directed strategy that increases their resilience to future stressors. It is this complexity between children's individual capacities to cope on their own and the capacity of their environments to provide care and support that needs further exploration.

## Literature review

There is very little research that explains children's patterns of coping following maltreatment that includes analysis of individual coping strategies, social supports and the constellations of human services many of these children access. Instead, research has typically focused on one of these three domains. For example, it is widely acknowledged that the more children experience multiple adversities, the more complex children's pathways into, through, and out of services, will be (Grimes *et al*. 2006). Delinquent children who have experienced neglect or abuse are well known to decline formal services and prefer individual coping strategies that may not fully address their psychological and behavioral problems (France *et al*. 2012; Losel & Farrington, 2012). Intra-personal coping strategies are less effective, though, if the child's exposure to stress remains high (Ungar, 2013).

Studies of children in foster care can help to illustrate the complex interaction between children's coping strategies, the supports they receive and the specificity of the adversity they experience. Pecora (2012) showed that, overall, the likelihood of foster children engaging with and succeeding at school is less than the national average when children are matched for age and demographic background. However, foster children showed better health outcomes and met national norms when permanency is pursued by child protection services, a strengths-based assessment is done, educational supports are provided, mental health problems are treated concurrently, individual life skills are taught, and children are motivated to complete high school. It is this combination of social supports, services and individual coping strategies that appear to help foster children cope with the multiple levels of stress associated with past abuse and out-of-home placement. It is not clear from Percora's work, though, how or why foster children choose intra- or interpersonal coping strategies, nor the catalysts that motivate a child to accept or reject supports and services when they are offered.

Whether willingly or not, children with complex needs and those exposed to multiple stressors tend to be, on the whole, engaged with many different services when those services are available. In a study of lifetime rates of mental health service use in a representative sample of 1706 children drawn from a large US metropolitan area (all participants were receiving services from alcohol and drug services, child welfare, juvenile justice, mental health and special education services for children with serious emotional disturbances) 87% were found to have used at least one outpatient service, 45% an inpatient service and 71% a school based service (Hazen *et al*. 2004). While these numbers are promising, similar studies also show that a minority of children who need services and have access to a comprehensive suite of services refuse interventions. Homeless youth, for example, despite exposure to serious maltreatment may identify mental health and substance abuse interventions as important but still infrequently engage with programming (Brooks *et al*. 2004). Youth with mental health needs related to past abuse and involved with the juvenile justice system typically receive far fewer interventions than young people with the same diagnosed disorders who are living with their families (Odgers *et al*. 2005; Rogers *et al*. 2006; Liebenberg & Ungar, 2014).

## Methodology

### Research design

To explore the processes by which children access different services and supports, we drew on qualitative data gathered from a purposeful sample of 608 youth who were engaged with service providers in five countries. In order to facilitate cross-site comparison of the data, a phased analysis was conducted, starting with ten interviews (two from each country, translated) followed by review in each country of the full set of qualitative interviews produced locally to confirm if the results from the cross-site analysis were trustworthy. All young people were part of a mixed methods study of risk, resilience and service use patterns among 7334 young people ages 12–19 in Canada, South Africa, New Zealand, Colombia and China. All youth resided in communities with high rates of violence, child maltreatment, or where a large number of residents had experienced dislocation due to forced migration and social marginalization. The qualitative component of that research involved a subsample of these youth who participated in follow-up interviews. Interviews included questions related to service use patterns, informal supports and their association with resilience in varied cultural contexts. The study also sought to understand, from the perspective of young people themselves, which are the most useful services and supports for mitigating risk and promoting well-being. The study met all ethical protocols at the five host institutions.

### Sample selection

The five research sites were chosen based on the criteria of maximizing variability and theoretical considerations related to the provision of services and supports around the world. Each of the five very different research sites included in this study introduced sufficient variation into the sample to compare the different ways services and supports are provided to young people who experience maltreatment in low-, middle- and high-income countries. Table 1 provides a description of the five research sites and the services and supports available to young people in each. Each research site was different from the others in regard to the risk factors facing young people. In this way, our purposeful sample provided a snapshot of a diverse range of interventions offered by services and supports, as well as young people's individual coping strategies.

**Table 1.** Description of the five research contexts

Location/setting	Service ecology
Eastern Canada	The Canadian research sites are in east coast urban and semi-rural communities with relatively high levels of social marginalization. Participants come from communities that have experienced historical racism, as well as poverty. They frequently have a family member with a mental illness or concurrent disorder (e.g. substance abuse and mental health challenges). Participants have good access to formal services and informal non-governmental supports. Youth programs are available to youth in their community and include universal access programming like sports and dance, and programs specifically for underprivileged youth. There is also a strong foster care system in place as well as education system. The education system provides access to guidance counselors and social workers
Beijing, China	As a rapidly developing metropolis, Beijing's urbanization has brought polarization between the rich and the poor, an influx of migrant workers, strained education resources, increases in divorce rates and unemployment. Participants experience both social problems and psychological challenges such as anxiety and depression and problem behaviors like bullying, smoking, alcohol use and internet addiction. Formal services are mainly provided by the school through individual counseling, group counseling, or student societies and teacher supports. The government has allocated funds for poor students and accompanying children of rural workers. Informal services include those provided by non-government organizations such as youth volunteers, college students' practical projects, enterprise charity and other efforts to engage young people in their communities
New Zealand	The sample was drawn from the 25% of New Zealand children who live in poverty and face significant disadvantage across a wide range of social and economic circumstances. Many of these youth come into repeated contact with the welfare, juvenile justice and mental health systems or find it a challenge to remain at mainstream schools. There are both formal and informal supports in place including foster care, community programs, educational programs and community organizations providing a range of sport and cultural activities. Youth are able to gain access to programming and supports through statutory welfare, justice, educational and mental health programs as well as programs provided by community, ethnic and tribal organizations
South Africa	The South African research sites were located in rural, resource-poor communities where youth are challenged by indigence, violence, high incidence of HIV and AIDS, and death- or migration-related loss of family and friends. Mainstream schooling is generally accessible to youth living in these areas, but other services (e.g. special education services, mental health and social welfare) are characterized by inequitable access and inadequate numbers of service providers. Typically, black youth in these areas rely on kinship support and other informal supports (e.g. church communities, teacher-initiated interventions) to survive
Medellin, Colombia	Though Medellin, a city of 3.5 million people, is a hub for financial, industrial and commercial services, it also has high rates of violence and homicides from drug trafficking, contract killings, gangs and poverty. Youth in our sample come from lower socioeconomic stratum living in marginal and poorly serviced contexts, with social inequality problems such as lack of access to health services, education, housing and recreation. Services are often provided by local churches or non-governmental organizations with limited capacity to provide universal access to care

Each research setting also had a Local Advisory Committee (LAC) comprised of mental health professionals, service providers (including child welfare workers and educators), researchers and community elders appointed based on the preferences of the local lead investigators. These investigators were asked to identify people who could comment authoritatively on services for young people at risk and help locate a sample of young people who met the study's inclusion criteria. Specifically, the study engaged young people who used multiple services and those who had been exposed to high levels of risk but showed resilience. The final selection of young people invited to participate in the full mixed methods study had all experienced substantial amounts of adversity (as determined by each LAC), with one or more of the following risk factors present in their lives: poverty, social dislocation, cultural disintegration, exposure to domestic or political violence, marginalization, a caregiver with a drug or alcohol addiction, family breakdown, mental illness of the child or a caregiver, exploitation as a child laborer, and physical or sexual abuse.

For the purpose of consistency across research sites, resilience was defined as the ability to ‘do well’ by the standards of each community. LACs identified young people who faced significant amounts of risk but showed resilience through engagement in protective processes such as remaining in school, avoiding delinquent or age-inappropriate behaviors (like early sexual initiation or school dropout), or were known to be making a positive contribution to their communities or families. Table 2 provides demographic information and a description of the risks facing each of the ten participants selected for cross-site comparison. The risks each faces are typical of the risks identified as common in each research site as detailed in Table 1.

**Table 2.** Participant information

Location/setting	Participant description^a^	Risk factors (brief selection identified by LAC)/referral source
Eastern Canada	Female, age 19 Aboriginal	Contact with Child Protection Services since age 8, mental health and addictions issues, homelessness/Referred by Child Protection Services
	Male, age 16 Aboriginal	Placed in foster care at age 11, learning challenges, violent behaviors/ Referred by Child Protection Services
Beijing, China	Female, age 16	Single parent family (father only), multiple household moves, poverty, father on disability allowance, conflict with extended family/Referred by school counselor
	Female, age 16	Uncle is primary caregiver (kinship adoption), extended family problems (Uncle's brother is disabled), poverty/Referred by school counselor
New Zealand	Female, age 17	Parents separated, father and mother struggle with addictions, father jailed while she was in his care, substance abuse, school problems/Referred by Child, Youth and Family Services
	Male, age 18	Parents divorced, disengagement from school, substance abuse, suicidal behavior and other mental health challenges/Referred by Child, Youth and Family Services
South Africa	Female, age 19 Black	Orphaned at age 10, placement in children's home, social stigma related to being an orphan/Referred by school teacher
	Male, age 17 Black	Father absent from home often for work, poverty, minor criminal behavior, school problems/Referred by school teacher
Medellin, Colombia	Female, age 18	Sexual abuse, separation from parents, substance abuse, had first child at age 17, poverty/Referred by Child Welfare Services
	Male, age 16	Both parents passed away by age 13, ended school in grade five, conflict with extended family/caregivers, substance abuse/Referred by Child Welfare Services

a Youth's ethnoracial background is the same as the dominant cultural group in each site unless otherwise noted.

In total, 608 qualitative interviews were conducted across the five research sites (Canada, *n* = 147; China, *n* = 48; Colombia, *n* = 64; New Zealand, *n* = 109; South Africa, *n* = 240). Each country lead was asked to consult with his or her LAC and choose at least two interviews that they felt were good examples of the diverse ways that young people coped under stress and the way local youth used supports and services. The referring services or supports are included in Table 2. In most cases child welfare workers, teachers and guidance counselors were the principal nominators of youth to the study based on prior knowledge of the youth's service use history and the youth's demonstrated capacity to cope in ways valued by their communities.

When necessary, interviews were translated into English, the common language for interaction between team members. Canada, South Africa, New Zealand and Colombia chose a boy and girl, China translated the interviews of two girls.

### Data collection

Participants were interviewed between 2011 and 2013 at least once in each research site and at a location convenient for them but where they would not be overheard by others. Interviews took place in schools, at home, in libraries and other public institutions, or sometimes outside when that seemed the most appropriate place. Interviewers were employed by the study and trained locally by the lead researcher in each site. Interviews in four of the five research sites followed a common interview guide (with inclusion of local questions). In South Africa, youth were interviewed using an interactive narrative methodology called the Mmogo^™^-method that was culturally appropriate (Roos, 2008). All interviews were digitally recorded and then transcribed.

### Data analysis

Data analysis strategies were informed by techniques to develop Grounded Theory and guided by Glaser and Strauss' constant comparative method (Glaser & Strauss, 1967). Both sensitizing concepts (those derived from previous studies to inform the present study) and indigenous concepts (those that arose directly from the data collected in the present study) were first used to identify themes. Attention was paid to individual events as well as the quality of the interactions between social supports and formal services. Previous reports on the quantitative findings from this study have informed an ecological interpretation of the data which explained both aspects of resilience (coping well under adversity) and service use patterns (such as service access and dependability) (Ungar *et al*. 2012).

#### Phase one

This first phase of the analysis began with one team (the Canadians) having three of its academic team members share the task of coding all local qualitative interviews. A Canadian version of a master codebook was then generated. Next, the ten interviews from all five sites were coded using this same codebook with modifications and additions as the findings emerged. A qualitative data analysis package, Atlas.ti 7.5, was used to facilitate analysis. Multiple readings of the interviews and discussions followed among members of the Canadian team until a tentative substantive theory was developed that responded to the research questions.

#### Phase two

The international team made efforts to deepen cross-site comparison of the data by inviting the lead researchers and, when possible, at least one community member from each LAC and one or more research assistants who helped with data analysis, to visit each of the five research sites over the duration of the project. The international team met annually to discuss findings and comment on emerging patterns in both the qualitative and quantitative data. Each site also produced a detailed description of the risk factors facing the youth in their sample and identified potential protective factors as a way of educating other team members to the contextual specificity of young people's use of services and supports.

#### Phase three

Once the Canadian team had agreement on the initial analysis of the ten interviews, the substantive theory they developed was written and shared verbally with researchers from each of the other country teams. Time was allotted for each team to provide their own analysis of the ten interviews and to consult with their LACs about the theory that was being generated.

#### Phase four

While the ten interviews were being analyzed together, data analysis was also taking place in each research site with the full set of qualitative interviews that had been done locally. Each country team was asked to assess the trustworthiness of the substantive theory developed across sites to see if local datasets refuted the theory or required the theory to be modified.

## Findings

**Fig. 1.**
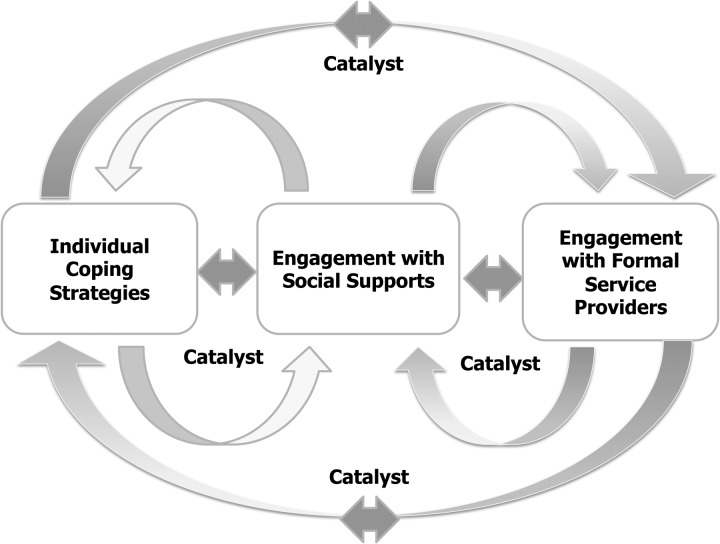
A Three Stage Reciprocal Coping Process.

To maximize their capacity to cope, and with recognition that services were reported by the youth to be either unavailable, excessively intrusive, or unreliable, participants described a three-phase reciprocal process that helped them improve their circumstances and experience resilience (see Fig. 1). In the first phase, youth rely on themselves to engage in cognitive and behavioral strategies that facilitate their adaption to stress. When these strategies are unsuccessful and the youth feel overwhelmed, they move to the second phase, engaging informal supports to cope better. In many cases, these supports are sufficient to mitigate exposure to risk. However, as was often the case, problems can be too complex for informal supports to address entirely. When failure is likely, youth are compelled to seek an alternative coping strategy that includes help from formal services where these are available. When services fail to meet a youth's needs, or youth become better able to cope on their own, they again enter a period of transience, moving from one type of coping strategy to another. They might rely on informal supports, or decline help altogether and use individual strategies to cope during the next crisis.

The search for negative cases in our data showed that the model depicted in Fig. 1 is not unidirectional. Youth reported complex patterns of navigation between these three phases, in some cases completely reversing the order, using just two of the three strategies, or using all three simultaneously. For example, the South Africa female participant explained that her teachers (who performed the role of a social support when they act outside their mandated role as formal educators) recognized that the girl needed more formal support when she was orphaned and called the social workers to help. Together, the teachers and the social workers compelled the girl to accept services. At that point, the need for a formal intervention was high and there was capacity among local providers to meet the girl's needs. During her stay at a children's home, however, she befriended a hockey player whose family informally adopted her for a while. When the placement broke down, the girl was returned to the children's home. Following these multiple dislocations, her explanation of her resilience is very individually focused. She explained that one should ‘rely on yourself’ and on God, ‘coz God is more trustworthy’ than informal supports and formal services. While each of our ten cases shows a different pathway through the possible coping strategies, together they point to the need to consider the process of transience as having multiple starting points and no conclusion.

Broader analysis of all 608 qualitative interviews by country teams confirmed the patterns identified during the cross-site phase of investigation. Only minor changes resulted, such as South African data showing more ambiguity in professional roles (teachers could provide many different types of informal and formal support depending on the needs of the young person). The New Zealand data helped the multi-country team see the potentially positive ways young people cope without service providers even in highly resourced service contexts.

While the three phases of self-reliance, engagement with informal supports, and use of formal services are simple descriptions of coping strategies, a young person's decision to move from one strategy to the next, or back again, was contingent upon the youth's exposure to risk and the fit between the resources available and the youth's needs (for example, the South African male reported being beaten by the police, and then again by his mother, leaving him to rely on himself for help when he needed it). The complexity of these negotiations is best demonstrated through case studies that show the nuanced points of decision-making young people experience.

### Navigation into and out of services in a context of abuse

Abuse, both physical and sexual, required youth to find ways to adapt to traumatizing events. For example, the Colombian female suffered a rape at the age of 12. According to the youth, her sister had arranged a meeting with a man, provided alcohol for the youth, and the rape followed. During her interview, the youth recalled the event and its continuing impact on her life: ‘Even though I don't remember, I think my sister got money for that, even though I don't remember it I think that is a damage that I will never…even with time passing by, even though right now well I don't have as much distress about that’. To cope in a dangerous situation like this the girl used drugs and alcohol to suppress troubling thoughts and feeling, with her substance abuse increasing as time went on to include the consumption of large amounts of cocaine: ‘Sometimes I would even snort three or four (grams), and I never suffered of an overdose’.

Our data suggest a complex relationship between risk factors like these and the coping strategies available to young people. While drug abuse was never advanced as the most desirable solution, it did appear as a default coping strategy when other options were perceived as inaccessible. In this sense, drug abuse was in some instances an atypical but contextual accessible and popular coping strategy. By the time the young Colombian woman was interviewed for this study, she had found more socially desirable ways of coping by linking to social supports. Her transience from individual coping to support-seeking behavior, like the transience of other participants, began with what we coded as a ‘moment of clarity’: ‘From one moment on I said: “I am silly, I'm making my life miserable by myself, for something that that man is not going to remedy, for something that I'm not able to remedy, that has already happened and I'm not going to make life miserable”’. This moment of clarity became a catalyst for the young Colombian to look for other sources of support to cope better with her sexual victimization and the breach of trust with her sister.

Like the young woman from Colombia, the male youth from New Zealand was also a victim of sexual abuse and showed similar coping strategies. Sexually abused by his father at a very young age, the boy's father was sent to jail and has not been a part of the youth's life since. The stressors at home piled up after the sexual violence was disclosed. The youth's mother fell into a severe depression, his sisters left home at an early age, and the youth was left to take care of his mother: ‘She got quite bad depression and anxiety. She got quite unwell. So I was pretty much looking after her and me since a young age’. The time spent providing care to his mother resulted in the youth missing a great deal of school and created problems for him when he did attend: ‘I had to take care of the household. Had to sort everything pretty much…when you have that much time off at school by the time you go back to school it's like an alien concept, so I didn't really fit in, started acting out a bit’.

The youth's time away from school not only created difficulties academically, but also socially. He had trouble connecting with the other students or forming positive relationships: ‘I don't really like have mates, I just sort of like have people that if they walk past then I'll say: hey, how's it going? Quite a lot of people know me, I don't know how but everyone knows me so – but I wouldn't say I have like mates…I don't like people my age. They annoy me. Too immature, yeah’. Much like the Colombian female, substance abuse became a maladaptive strategy for the New Zealand male to deal with his early abuse. Interestingly, though, just as the Colombian youth had a moment of clarity, this New Zealand youth also realized that he had to stop using drugs. When asked why he stopped, he said: ‘I want to try and make as best a life as I can for myself and I don't see drugs and alcohol doing that for me so’.

Given the youth's access to a number of child and youth workers and social workers who were employed by formal service agencies, he was able to navigate his way into a training course. He also began rebuilding a car with his uncle who was a mechanic. In this case, reciprocity meant not only a change in the young man's cognitive and behavioral coping strategies, but also collaboration between formal service providers and the informal supports the youth needed. Specifically, service providers helped arrange for the youth to live with his aunt and uncle, a housing situation that he reported was working well. Other informal supports included a girlfriend with her own history of abuse.

Though the network of informal supports that were bridged by formal service providers helped the youth stabilize, he still suffered from what he described as severe depression and experienced multiple admissions to hospital. However, the situation could, according to the youth, have been much worse without the supports.

The logic of the transitions from one coping strategy to the next can be understood in relation to the resources available to the young person and the young person's own values and beliefs. For example, one of the young women in China whose mother had died while giving birth to her had experienced a great deal of instability. Her maternal grandparents had threatened to kidnap her and the girl's father had been forced to live with his parents. Later, when the girl's paternal grandparents died, there were arguments over the inheritance that led to the youth and her father moving again. She explained: ‘He didn't want to argue with them so we moved out of grandma's house. So far, my dad and I have had to change places six times to avoid them. It's better now because they don't come looking for us, but they used to threaten my dad’.

Despite this turmoil, the youth said that she had been able to do reasonably well in life because of the availability of strong formal and informal supports. Emotionally, she was fortunate to have had a peer group she could rely upon, as well as several adults with whom she engaged at school: ‘You know, there are more and more people who help me, and more and more people who watch me grow up; and that gives me the will to carry on. I think that I would be pulled back into sadness and gloom if I were to stop talking to people’.

Her friends, classmates, and teachers all played a large role in supporting her during difficult periods of transition that included multiple moves. As in other case studies, one of the girl's teachers became a particularly important resource: ‘She was just so great! She knew my entire family by heart. Those times where my uncle came to school to take me away, she would prevent any harm from happening to me’.

As each example shows, a child's individual capacity to cope, his or her social supports, and the child's service ecology influence a young person's coping strategies. Even when services are available, however, personality factors, values and community pressures to conform influence a young person's pattern of coping. Where resources are largely unavailable or provided in ways that are not well matched to a child's needs, the tendency is for young people to resist transitioning from individual coping strategies to engagement with informal or formal helpers.

## Discussion

Further analysis of the entire dataset that focused on youth who had experienced the loss of a primary caregiver or multiple dislocations between communities showed similar patterns of complex reciprocal navigation between the three phases of coping in each country. Given these results across a heterogeneous, purposefully selected multi-country sample, our findings suggest that young people who have been exposed to serious adversity in different contexts show similarities in the processes they use to cope. Broadly speaking, young people engage in individually focused coping strategies (cognitive and behavioral), seek help from social supports, or accept formal services from institutional providers. As one strategy succeeds or fails, other strategies become more desirable. However, the transience from one of these three types of coping to another usually results from a catalyst moment in which a set of coping strategies requires change. As our data show, and as found in other studies, young people use different coping strategies that frequently overlap or complement each other depending on whether they have access to services and supports that function well enough to meet their needs (Hazen *et al*. 2004; Ungar *et al*. 2012).

In general, we observed a tendency for young people to move from individual coping strategies to informal social supports and then formal services. In instances where this pattern was different (a negative case example like the South African female participant), there was a strong likelihood that formal services had been introduced too quickly. Youth rejected help from formal service providers when they perceived that less intrusive solutions were available.

Our goal is not to identify any one fixed pattern of transience, but instead to note the fluidity of the movement between all three types of coping. We were particularly interested in how young people in contexts without many formal services (e.g. Colombia, South Africa and China) still managed to cope well. For example, young people in low- and middle-income countries seemed more likely to rely on extended kinship networks or experience blurred boundaries with professionals like teachers and social workers who would offer supports that were beyond their professional mandates.

Given the creative (and necessary) use of social supports by young people living in poorly resourced environments, we wondered if the presence of formal services in high-income countries such as Canada and New Zealand skew a youth's way of coping under stress? A decision to engage with services was not simply a matter of a service being needed. In many cases, a service might have been useful but in a low-resource community was unavailable or inaccessible.

Contrariwise, in higher resource communities, services could pre-empt opportunities for social supports to offer effective help to a child facing adversity. Formal services could undermine personal agency. In other words, personal coping strategies, social supports and formal services are resources that conflict depending on an individual's or community's values and resources.

Our findings also show that when formal services are not available, informal supports can take on multiple roles (e.g. teachers become case workers) or youth will rely more heavily on socially desirable individual coping strategies (e.g. religious faith) as they have few other ways to cope with stressors. In fact, we note that in contexts where there are formal services and they are offered too quickly, youth who show resilience may resist interventions in order to maintain a sense of self-worth and personal agency.

## Limitations

Given that our research was qualitative, we cannot generalize the results beyond these specific case studies or these research sites. Future research will be required to see if these patterns of transience are transferable to other youth populations. Even with a relatively large qualitative sample in each research site, the three phase substantive theory we have outlined may only be relevant to young people who face a significant number of risk factors similar to those youth purposefully selected for this study. Though we found common patterns, we wonder if a different set of interviews had been selected by the LACs whether our analysis would have led to slightly different findings.

## Implications for interventions

For youth who have experienced higher levels of chronic and acute stress there has been a great deal of attention paid to providing formal services in low-, middle- and high-income countries (when these resources can be made available by governments or non-governmental organizations). For example, it has been suggested that even in highly stressed poorly resourced countries formal mental health interventions are necessary for former child soldiers, child refugees and children who have been exposed to sexual violence (Betancourt, 2012; Tol *et al*. 2013). None of these efforts are contradicted by our findings, though the reluctance of some young people with histories of maltreatment, dislocation or social marginalization to engage with formal services may indicate that they experience themselves as more resilient when they resist interventions. It is particularly important to note (with regard to treatment planning) that some of the youth in the present study who had experienced potentially traumatizing events hesitated to accept services or engage with social supports until their own solutions had become unsustainable or self-harming. In other instances, like that of the South African girl where teachers and friends proved unreliable, self-reliance became the default coping mechanism despite initial acceptance of support from others.

Learning from this data, there are lessons for treatment planning in low- and middle-income countries, high-income countries and all countries. In low-income countries where human services are often poorly resourced, caution is needed before professionalizing services at the expense of broader prevention efforts. Helping young people themselves to cope on their own, or helping them identify and engage with social supports, may be just as effective as introducing costly formal services for children with acute mental health problems. Participants in this study showed remarkable capacity to overcome adversity by making the best use possible of non-formal supports. Programs that improve parenting capacity, for example, may be just as effective at building young people's resilience as trauma-informed care delivered directly to children themselves (Gewirtz *et al*. 2008).

In high-income countries, our findings suggest a treatment paradox. The provision of high-quality services may undermine young people's engagement with their own natural supports. Furthermore, when young people experience professional services as unresponsive to their needs, youth may turn more to themselves than to social supports, in part because of an ideology of individualism. In both cases, youth from high-income countries showed us that part of the role of professional helpers must be to build bridges between young people and their natural support systems if we are to provide youth with the largest number of options to nurture and sustain resilience.

For all young people, from low-, middle- and high-income countries, a better way of structuring interventions may be to consider a few important principles. First, whenever possible, offer the least intrusive solution to young people's problems. Universal access programming can bolster individual coping (e.g. school-based self-regulation training or information on the long-term negative impact of harmful involvement with substances). Second, even when formal interventions are possible, providers of children's services should help young people find the social supports they need to cope after treatment ends, or if they experience treatment as ineffective. Third, and as a corollary to the second principle, individual coping strategies should be encouraged as part of a process of transience. Though service providers tend to think in terms of what they can do for young people exposed to high levels of adversity, young people seem to prefer the strategic use of individual coping strategies, social supports and formal services one after the other or at the same time. The more service providers are patient with young people and let them choose when they want treatment the more likely treatment will be seen as helpful. Together, these three principles suggest that professional services need to be both easily accessible and provided in ways that respect children's rights to control the decisions that affect them.
